# Numerical and experimental investigations of the flow–pressure relation in multiple sequential stenoses coronary artery

**DOI:** 10.1007/s10554-017-1093-3

**Published:** 2017-02-20

**Authors:** S. Li, Cheng Chin, Vikas Thondapu, Eric K.W. Poon, Jason P. Monty, Yingguang Li, Andrew S.H. Ooi, Shengxian Tu, Peter Barlis

**Affiliations:** 1 Department of Mechanical Engineering, Melbourne School of Engineering, The University of Melbourne, Melbourne, VIC Australia; 2 Department of Mechanical Engineering, The University of Adelaide, Adelaide, SA Australia; 3 Department of Medicine, Faculty of Medicine, Dentistry & Health Sciences, The University of Melbourne, Melbourne, VIC Australia; 4 Division of Image Processing, Department of Radiology, Leiden University Medical Center, Albinusdreef 2, 2300 RC Leiden, The Netherlands; 5 School of Biomedical Engineering, Biomedical Instrument Institute, Shanghai Jiao Tong University, Shanghai, China

**Keywords:** Flow–pressure relation, Computational fluid dynamics, Fractional flow reserve

## Abstract

Virtual fractional flow reserve (vFFR) has been evaluated as an adjunct to invasive fractional flow reserve (FFR) in the light of its operational and economic benefits. The accuracy of vFFR and the complexity of hyperemic flow simulation are still not clearly understood. This study investigates the flow–pressure relation in an idealised multiple sequential stenoses coronary artery model via numerical and experimental approaches. Pressure drop is linearly correlated with flow rate irrespective of the number of stenosis. Computational fluid dynamics results are in good agreement with the experimental data, demonstrating reasonable accuracy of vFFR. It was also found that the difference between data obtained with steady and pulsatile flows is negligible, indicating the steady flow may be used instead of pulsatile flow conditions in vFFR computation. This study adds to the current understanding of vFFR and may improve its clinical applicability as an adjunct to invasively determined FFR.

## Introduction

Fractional flow reserve (FFR) has been generally accepted as the gold standard for detecting functional significance of intermediate coronary stenoses [3]. It is defined as the maximum achievable myocardial blood flow in the presence of a coronary stenosis divided by normal maximum flow. FFR can be derived from ((*P*_*a*_ – ∆*P*)/*P*_*a*_) using aortic pressure, *P*_*a*_, and the pressure drop $\Delta{P}$ along the lesion measured during invasive coronary angiography under hyperemic flow condition [10, 17]. Although FFR-guided Percutaneous Coronary Intervention (PCI) significantly improves the clinical outcome and reduces the mortality rate of multivessel coronary artery disease (CAD) compared with angiography-guided intervention [3], practical drawbacks of measuring FFR including its invasive nature, cost, and the need to induce hyperemia, have limited its clinical applications [9]. As such, virtual FFR (vFFR) has sought to replace invasive FFR for physiological assessment of severity of intermediate coronary lesion in arteries. Virtual FFR utilizes computational fluid dynamics (CFD) to compute the hemodynamics on a reconstructed patient-specific coronary artery model using either coronary angiography, intravascular ultrasound or optical coherence tomography. The data is used to compute FFR without a pressure guidewire [11]. However, the optimal approach of CFD simulations for the computation of vFFR is still under consideration. Although pulsatile flow is considered to be the most accurate model to simulate coronary blood flow in circulation, the computational expense with regards to time and hardware requirements restricts its clinical applicability. Steady-state CFD simulation has been considered to be a more practical approach given that it requires much less computational time. Tu et al. [13] and Poon et al. [12] have shown close agreement in the vFFR results between steady and pulsatile flows. However, this result might not be applicable for more complex geometry model beyond a single segmental stenosis. Li et al. [7] have investigated hemodynamics of the outgoing flow through side coronary branches and stated that true anatomical tree model that takes into account the flow through side branches must be used for accurate computational fluid dynamics analysis in coronary artery. However, the hemodynamic interaction between serial coronary stenoses within a single coronary artery, which is commonly present [8], has not yet been assessed. The presence of serial stenoses increases the difficulties in assessing the hemodynamic significance of each lesion that may have an effect on PCI decision.

As mentioned above, the knowledge of pressure drop over stenosis is required to calculate the FFR. It is thus important to investigate the pressure drop performance in stenotic coronary artery to assist in vFFR calculations. Young et al. [17] examined the relationship between the flow velocity and pressure drop across one single stenosis. They have derived an equation connecting the severity of a stenosis, mean flow velocity and the corresponding pressure drop. However, the predicted pressure drop is only valid for a single coronary lesion. Pijls et al. [10] investigated the hemodynamic change in the presence of several stenoses within one artery. They confirmed that the hemodynamic significance of each stenosis is influenced by other stenoses and emphasised the importance of using pressure drop to evaluate the hemodynamic significance of each lesion. However, the relationship of flow–pressure over serial stenoses remains to be investigated. Having a clear understanding of the flow–pressure relation in a coronary artery with multiple plaques will essentially help assess the difficulties in the generation of hyperemic flow for vFFR calculations.

This study aims to investigate the flow–pressure relationship in multiple sequential stenoses coronary artery. Both high fidelity experimental and CFD methodologies will be used on an idealised serial stenotic coronary artery model. A realistic physiological pulsatile and steady-state velocity profiles are applied to evaluate if there is any difference between data obtained using pulsatile and steady state inlet conditions if only the average quantity is of interest. The study provides a suggestion of the optimal approach for vFFR calculations.

## Methodology

### Geometry

**Fig. 1:**
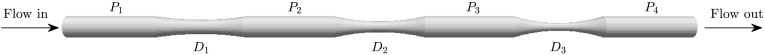
An idealized three-dimensional axisymmetric coronary artery model with multiple coronary lesions in series. Diameters of stenosis (DOS) from left to right are: *D*_1_ = 2 mm, *D*_2_ = 1.5 mm and *D*_3_ = 1 mm, respectively. Pressure measurements are taken at the locations *P*_*1*_, *P*_*2*_, *P*_*3*_ and *P*_*4*_

Figure 1 shows the schematic diagram of a diseased coronary artery with multiple stenoses in series. To simplify analysis, the diseased coronary artery is modeled as a rigid axisymmetric cylindrical tube with axisymmetric narrowing. The diseased coronary model has a segment length of 40 and 3 mm reference diameter, *D*, which corresponds to the normal diameter of most relevant coronary arteries in general. We considered three coronary stenoses in series, each lesion has a length of 10 mm that is typically required for interrogation of the functional significance of coronary stenoses. The diameters of stenosis (DOS), from proximal to distal, are *D*_1_ = 2 mm (non-obstructive stenosis that is unlikely to cause ischemia.), *D*_2_ = 1.5 mm (the typical stenosis for interrogation of the functional significance of coronary stenoses.) and *D*_3_ = 1 mm (the severe stenosis that will be stented without FFR assessment.), respectively. A further 10 mm segment is added to both proximal and distal ends of the coronary model.

### Experiment configuration

**Fig. 2:**
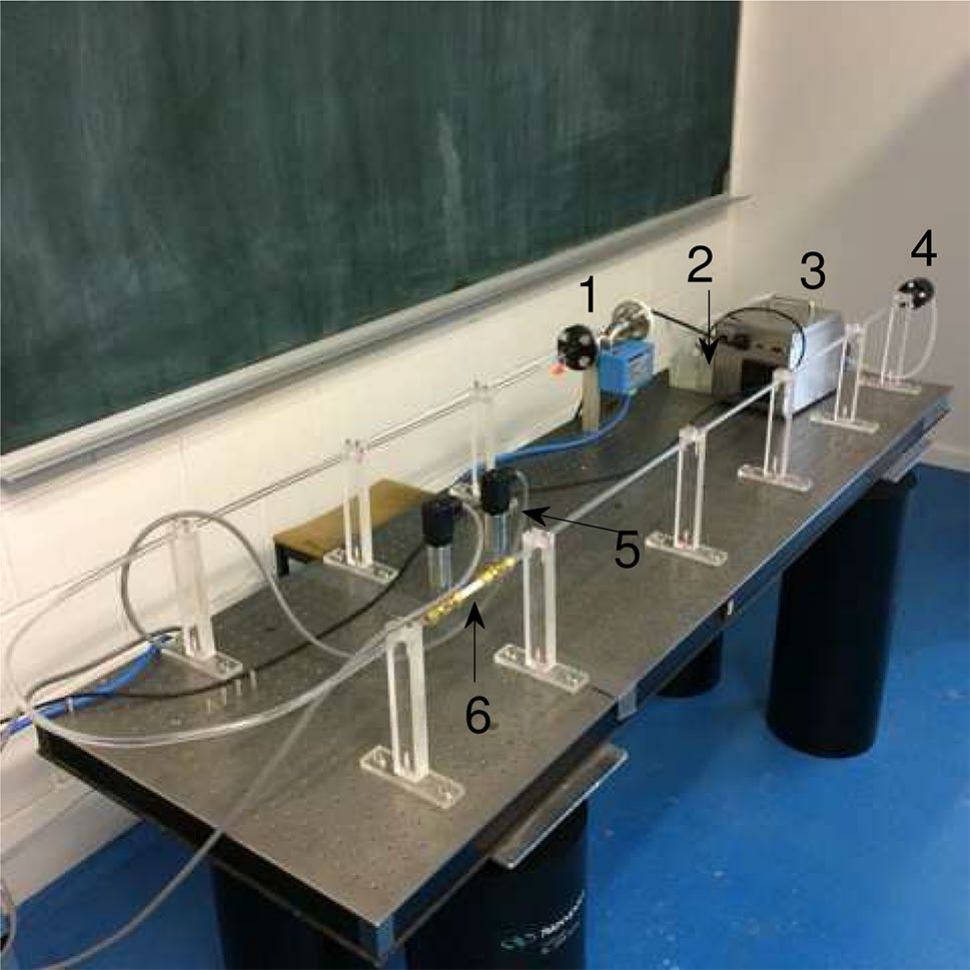
Experimental setup: pulsatile flow for multiple stenosis. (*1*) flow meter; (*2*) reservoir; (*3*) Ismatec pump; (*4*) settling chamber; (*5*) Omega pressure transducer; (*6*) 3D printed phantom multiple stenosis model

**Fig. 3:**
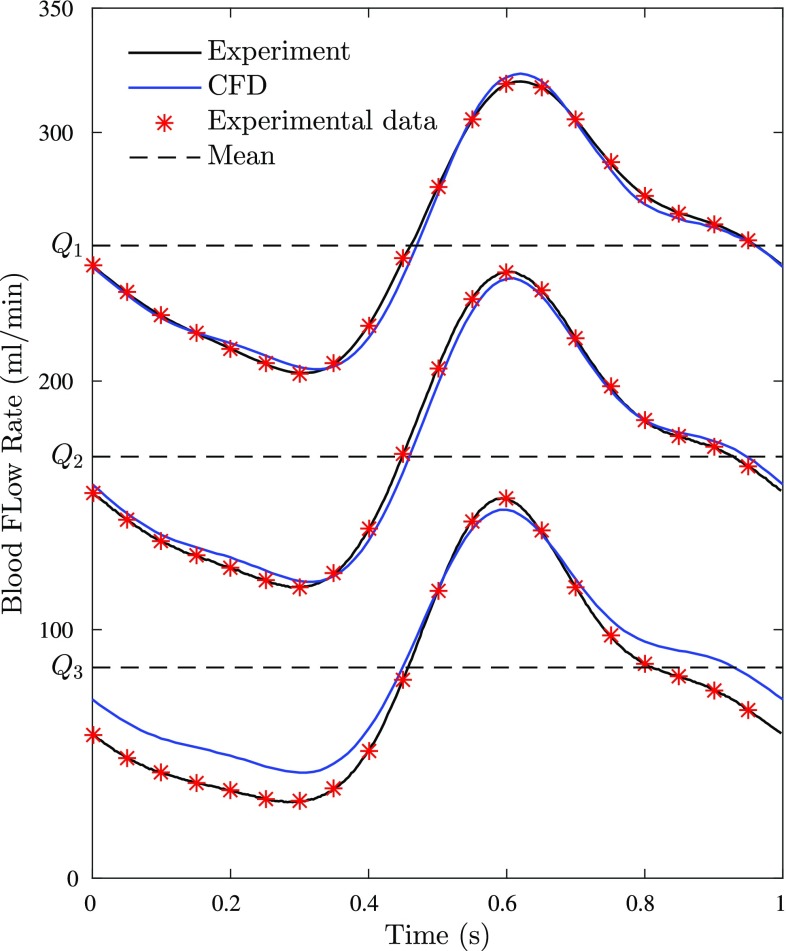
The physiological pulsatile inlet velocity boundary conditions with mean flow rate ${Q}_{1}$ = 254.5, ${Q}_{2}$ = 169.6 and ${Q}_{3}$ = 84.8 ml/min

The experimental setup is shown in Fig. 2. It consists of the following components: (1) flowmeter (ELITE Series Coriolis Flow meter, CMF050M), (2) water reservoir, (3) fluid pump (Ismatec, BVP-Z), (4) settling chamber, (5) pressure transducers (GE Druck—UNIK5000 series), (6) 3D printed multiple stenosis model (Objet Eden260VS, Stratasys) and (7) data acquisition board (DT9832, Data Translation). The data acquisition board acquires data from the pressure transducers and the flow meter at a sampling rate of 2 kHz. The stenosis model is approximately 1.2 m from the exit of the settling chamber to ensure a fully developed laminar Womersley velocity profile. The pulsatile waveform, as shown in Fig. 3, obtained from Huo and Kassab [5] was decomposed using Fast Fourier Transform. The input signal is reconstructed using the first 10 Fourier modes and is proven to accurately represent the waveform considered. This corresponding waveform is later discretised into a voltage–time signal with 800 samples and entered into the Ismatec pump through data acquisition board, with an in-house written m-file in MATLAB 2014a (MathWorks Inc., Natick, Massachusetts, USA). The flowmeter and the pressure transducers have a measurement error of ± 2 and ± 1% respectively. Two pressure transducers are used to measure the pressure difference at any two locations (P1 to P4 as indicated in Fig. 1). The fluid used in the experiments is a mixture of 50% glycerine and 50% distilled water to obtain a viscosity of μ = 0.004 Pa s to match that of blood. The model used in the experiment is scaled up twice that of the simulations (i.e. the diameter is 6 mm). The mean Reynolds numbers is matched with the simulations to ensure that similar flow physics is obtained between the experiments and simulations.

### Computational fluid dynamics

We employed an identical waveform as the in-vitro setup for the CFD studies. To minimize computational effort and disturbance at the inlet, a Womersley velocity profile [16] with the mean flow rate *Q* = 84.8, 127.2, 169.6, 212.1, and 254.5 ml/min was prescribed at the inlet (see Fig. 3, only waveforms with mean flow rate *Q*_1_ = 254.5, *Q*_2_ = 169.6 and *Q*_3_ = 84.8 ml/min are plotted). The lowest flow rate selected corresponds to reported resting blood flow rate while the highest mimics hyperemic flow. Outlet pressure and wall boundary conditions were specified as zero. Outflow (fully developed flow) condition was applied at the outlets, whereas non-slip condition was applied at the lumen wall.

Numerical simulations of pulsatile flow through a series of stenoses artery model were performed in the commercial software ANSYS FLUENT 15.0 (ANSYS, Inc.) by solving the incompressible Navier–Stokes equations:

1 $$\rho \left( \frac{\partial \mathbf{u}}{\partial t}+\mathbf{u}\cdot \nabla \mathbf{u} \right)=-\nabla P+\nabla \mu \cdot (\nabla \mathbf{u}+\nabla {{\mathbf{u}}^{T}}),$$

2 $$\nabla \cdot \mathbf{u}=0.$$

Equations (1) and (2) were implemented in each cell and nonlinear partial differential equations were solved simultaneously. Blood was modeled as incompressible Newtonian fluid. A blood density of ρ = 1060 kg/m^3^ and viscosity μ = 0.0035 Pa s were applied. The SIMPLE pressure–velocity coupling method was used. Second order scheme was chosen for the pressure discretization and second order upwind scheme for the momentum equations. The residual error convergence threshold was set as 0.00001. For the unsteady simulations, 300 time instances were saved in each cycle.

## Results

**Fig. 4:**
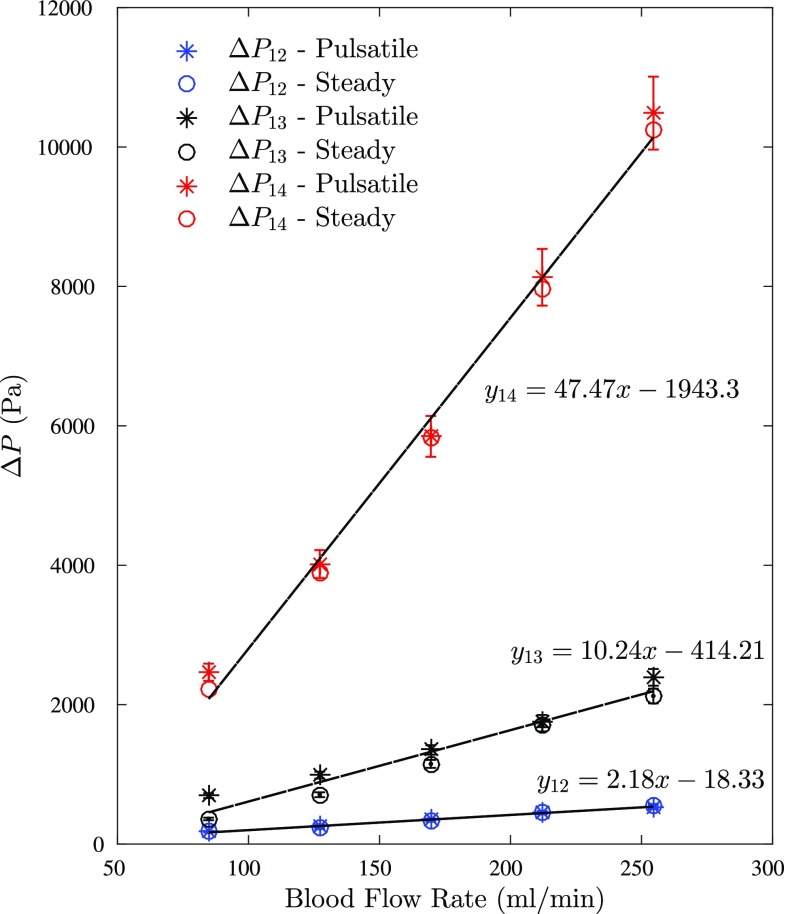
The pressure drop measured across different numbers of stenosis in the vessel with flow rate change in experiments; Δ*P*_12_: the pressure drop between the first stenosis and the inlet, Δ*P*_13_ : the pressure drop between the first two stenoses and the inlet, Δ*P*_14_: the pressure drop between the inlet and the outlet. The experimental error bars are ±5%

**Fig. 5:**
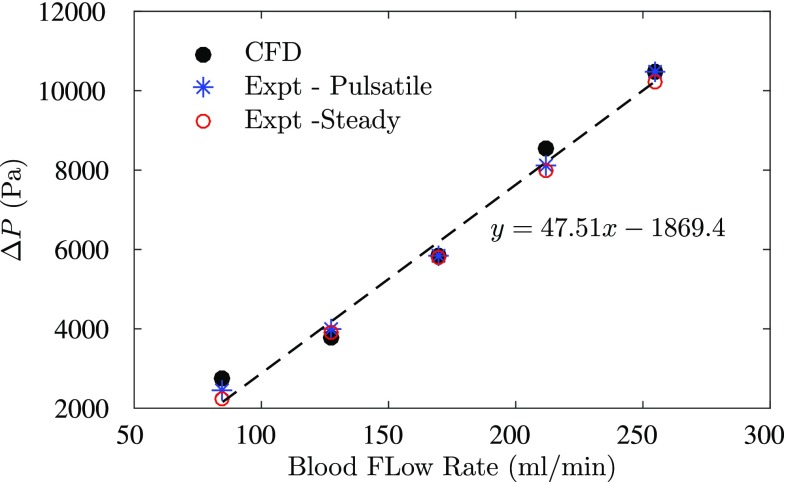
The pressure drop across the entire vessel generated from both experimental and numerical studies

As shown in Fig. 3, the pulsatile waveform prescribed in the experiments and numerical simulations are in good agreement with each other for different flow rates. Figure 4 shows the pressure drop with flow rate change as a result of presence of multiple sequential stenoses across one coronary vessel. The results were generated under both pulsatile and steady inlet boundary conditions in the experiments. The pressure drop was measured between one, two and three lesions, respectively. The details of experimental measurements are displayed in Tables 1 and 2. As the results shown, a positive correlation between pressure drop and flow rate regardless the location of the pressure drop measurements is observed. Also, at the same flow rate, increasing the amount of stenoses between the pressure drop measurements can result in a higher pressure drop. The results follow the same trend as mentioned before in both steady and pulsatile flows. Meanwhile, it is found that the results in both steady and pulsatile are generally close to each other at each flow rate. Figure 5 displays the pressure drops measured over three stenoses in both experiments and numerical simulations. The CFD results were obtained in the pulsatile flow only. The experimental and CFD results are in good agreement at each flow rate with only approximately 5% difference as indicated in Fig. 5.

**Table 1:** Pressure drop over multiple sequential stenoses in the vessel for the steady flow

Velocity(m/s)	Flowrate (ml/min)	Δ*P*_12_ (Pa)	Δ*P*_13_ (Pa)	Δ*P*_14_ (Pa)
0.2	84.8	174	360	2216
0.3	127.2	255	708	3897
0.4	169.6	337	1150	5819
0.5	212.1	447	1700	7975
0.6	254.5	548	2124	10,240

**Table 2:** Pressure drop over multiple sequential stenoses in the vessel for the pulsatile flow

Velocity(m/s)	Flowrate (ml/min)	Δ*P*_12_ (Pa)	Δ*P*_13_ (Pa)	Δ*P*_14_ (Pa)
0.2	84.8	181	694	2465
0.3	127.2	269	989	4017
0.4	169.6	351	1351	5848
0.5	212.1	452	1764	8131
0.6	254.5	529	2392	10,486

## Discussion

The results reveal a strong linear correlation between the pressure drop and flow rate regardless the amount of stenoses across which the measurements of pressure drop were taken under both pulsatile and steady inlet boundary conditions. However, the slope of the line indicates that pressure drop increases with the number of stenoses. Likewise, present CFD results are highly in line with the experiment results. Meanwhile, the results in Figs. 4 and 5 have shown that the difference between the steady and pulsatile flows are negligible with the pressure drop generated from pulsatile flow only slightly higher than the steady flow results.

The linear relationship between pressure drop and flow rate of multiple sequential stenoses within one coronary artery implies the simplicity of hyperemic flow production in vFFR calculation that enlightens the clinical applicability of vFFR. Being able to estimate the hyperemic flow is important for the calculations of vFFR as it allows one to mimic the realistic circumstance for invasive fractional flow reserve measurement. Flanigan et al. [4] have stated in their study that the pressure drop along stenosis in pulsatile flow is contributed by three mechanisms: laminar friction loss, turbulence generated in the post-stenotic diverging section and inertial forces due to pulsation. This relationship can be characterized by the following governing equation [17]:

3 $$\Delta P=\frac{{{K}_{v}}\mu }{D}V+\frac{{{K}_{t}}}{2}{{\left( \frac{{{A}_{0}}-1}{{{A}_{1}}} \right)}^{2}}\rho |V|V+{{K}_{u}}\rho L\frac{dV}{dt},$$

in which *K*_*v*_, *K*_*t*_ and *K*_*u*_ are experimentally derived friction, turbulence and pulsation coefficients to represent their proportional contribution to Δ*P, ρ* = mass density, *V* = flow velocity in the unobstructed vessel, *μ* = viscosity, *A*_1_ = cross-sectional area of stenosis, *A*_0_ = cross-sectional area of unobstructed artery of diameter D and *L* = length along the pressure drop measurements.

The formula reveals that the linear relationship between pressure drop and flow rate is attributed predominately by the friction loss term. It has been specified in [4] that significant pressure drop only occurs when the critical area of the lumen has been met, which is between 70 and 90% of area reduction depending on the flow rate, according to the vivo and vitro results in [4]. Passing the critical threshold value means a significant amount of turbulent energy generated in the lesion are dissipated in the post-stenotic region that dominates the change of pressure proportional to the square of flow rate. In our model, the maximum area reduction in the third stenosis is 89% and may not satisfy the critical area condition. The influence of turbulent energy losses is hence restricted. In addition, cumulative effects of serial stenoses can be equivalent to an elongated length of the artery contributing to an increase of friction coefficient in Eq. (3). Accordingly, the gradient of flow–pressure relation increases with an incremental amount of stenoses between the locations for pressure measurements as observed in Fig. 4.

The investigation in steady and pulsatile flows has further demonstrated that it is possible to obtain respectable accurate vFFR calculation with steady-state flow. The velocity gradient of the pulsatile flow characterized by Womersley number,

4 $$\alpha =\frac{D}{2}{{\left( \frac{2\pi \rho }{T\mu } \right)}^{\frac{1}{2}}},$$

is only 1.93 in this study. The Womersley number is relatively small in comparison with that of other parts of cardiovascular system (ascending Aorta − 13.2, carotid artery − 4.4, etc. [2]) that implies minor effects of the pulsation of a physiological cardiac cycle on the pressure drop. The study suggests that using steady flow is an optimal computational method of vFFR as it can speed up the calculation to a great extent without compromising the accuracy.

The comparison between CFD and experimental results sheds light on the accuracy, sensitivity and reliability of modelling vFFR in multiple sequential stenoses coronary artery. Likewise, the applicability of using steady-state flow and simplified hyperemic flow modelling indicates the possibility and reliability of fast vFFR computations that significantly reduces the computational time without compromising the accuracy. Accordingly, this will increase the utility of vFFR in the clinic.

This study is limited by the idealized geometry model. Lesions with more complex morphology might lead to more flow turbulence, altering the pressure–flow relation. In addition, we did not study very tight lesions where more pressure drops might be expected due to a greater likelihood of laminar–turbulent transition in the post-stenotic region. Finally, our CFD simulations ignored the elastic property of vessel wall and the dynamic courses of lumen geometry of coronary artery during heartbeat. Thus, the linear relation between flow and pressure drop as observed in this study might not completely represent the in-vivo situation. Earlier studies have modeled the compliance vessel using fluid structure interaction formulation [1] and investigated in experiments the elastic material property [15]. Future in vivo studies using patient-specific artery models that incorporate the compliance characteristic of the artery wall and more complex lesion morphology might provide further insights. Nevertheless, simplified approaches for fast computation of vFFR in vivo have been proposed recently and early validations showed promising results [13, 18].

## Conclusion

A linear correlation between pressure drop measured over multiple sequential stenoses and flow rate was observed. CFD analysis was validated to have a good agreement with experimental results that shed light on the accuracy of the vFFR calculation. Meanwhile, steady flow has been demonstrated as a reliable replacement of pulsatile flow for vFFR calculation as it shortens the duration of computation while yields the respectful accuracy. The conclusions drawn from the study have further enlightened the clinical applicability of vFFR.
